# Exploring Pathways of Socioeconomic Inequity in Vegetable Expenditure Among Consumers Participating in a Grocery Loyalty Program in Quebec, Canada, 2015–2017

**DOI:** 10.3389/fpubh.2021.634372

**Published:** 2021-08-02

**Authors:** Yu Ma, Cameron McRae, Yun-Hsuan Wu, Laurette Dubé

**Affiliations:** ^1^Desautels Faculty of Management, McGill University, Montreal, QC, Canada; ^2^McGill Centre for the Convergence of Health and Economics, McGill University, Montreal, QC, Canada; ^3^Department of Public Health, China Medical University, Taichung, Taiwan

**Keywords:** vegetable consumption, nutrition-sensitive agriculture, dietary guidelines, healthy food system, consumer behavior, food retailing

## Abstract

Vegetable consumption remains consistently low despite supportive policy and investments across the world. Vegetables are available in great variety, ranging in their processing level, availability, cost, and arguably, nutritional value. A retrospective longitudinal study was conducted in Quebec, Canada to explore pathways of socioeconomic inequity in vegetable expenditure. Data was obtained for consumers who participated in a grocery loyalty program from 2015 to 2017 and linked to the 2016 Canadian census. Vegetable expenditure share (%) was examined as a fraction of the overall food basket and segmented by processing level. Panel random effects and tobit models were used overall and to estimate the stratified analysis by median income split. Consumers allocated 8.35% of their total food expenditure to vegetables, which was mostly allocated to non-processed fresh (6.88%). Vegetable expenditure share was the highest in early winter and lowest in late summer. In the stratified analysis, the low-income group exhibited less seasonal variation, allocated less to fresh vegetables, and spent more on canned and frozen compared to the high-income group. Measures of socioeconomic status were all significant drivers of overall vegetable consumption. Consumers with high post-secondary education in the low-income group spent 2% more on vegetables than those with low education. The complexity of observed expenditure patterns points to a need for more specific vegetable consumption guidelines that include provisions by processing level. Implications for education, marketing, intersectional policies, and the role of government are discussed. Governments can scale present efforts and catalyze health-promoting investments across local, state, national, and global food systems.

## Introduction

Fruits and vegetables, in all their diversity, are the core targets of many nutrition and policy interventions implemented across the world, and yet, most individuals fail to meet recommended levels of their intake. Fruit and Vegetable Consumption (FVC) remains consistently lower than the WHO-recommended daily intake of 400 g per day (excluding potatoes and starchy tubers) (1–3). Global estimates for mean per capita consumption are 208.8 grams per day for vegetables and 81.3 g per day for fruits (4), which are especially troubling given the protective effects of FVC for the prevention of diet related non-communicable diseases (NCDs) like cardiovascular disease, cancer, type 2 diabetes, obesity, dental diseases, and osteoporosis (1, 5, 6). Each year, 1.5 million deaths globally are attributed to low vegetable consumption and another 2.4 million are attributed to low fruit consumption (6). In addition to its impacts on health and livelihood, low FVC also has a substantial impact on the economy, with some countries like Canada estimating the economic burden to be over CAD 3.3 billion each year, of which 30.5% is associated to direct healthcare costs (7). To address this gap, most countries have adopted nutrition guidelines reflective of global standards, while concurrently tailoring their implementation to fit the foods of local regions. As a result, great variability is present in regards to the type, or processing level, of fruit or vegetable recommended (8).

Global nutrition recommendations have been guided by outcomes stemming from the joint FAO/WHO “Promotion of Fruit and Vegetables for Health” (PROFAV) initiative that started in 2003. For example, the Kobe Framework for national and sub-national level nutrition interventions was developed to improve population health and farmer's income via higher FVC (9). However, the guidelines stemming from this initiative, as well as those implemented by countries around the world, fail to define consumption guidelines for specific varieties of fruit or vegetable (e.g., citrus fruits, berries, leafy greens) or processing levels (e.g., fresh, prepared, frozen, or canned), and instead, broadly promote an overall intake of 400 g of unspecified fruits and vegetables (1). Tied to outcomes from the Kobe workshop in 2004, “variety is important” and “fresh is better” phrases have been encouraged in promotional messaging (2). Some countries have actively engaged into awareness and advocacy with a strong focus on fresh fruits and vegetables (8, 10).

Regardless of the intensity of policy or other efforts along the farm-to-plate value chain, vegetable consumption varies largely within and across geographies, and is reflective of variances in individual and collective psychosocial, cultural, economic, and environmental determinants (11). In countries like Canada, for instance, the recently revamped food guide devoted half the plate to fruits and vegetables without specification of processing level; however, only imagery of fresh fruit and vegetables were included in communication materials (10). Meanwhile, Brazil considers canned fruits and vegetables as processed foods and advises only for fresh, frozen, or dried, and Australia only advises against added sugars, salts or fats in any fruit or vegetable product regardless of level of processing (8). Beyond geographical differences in the implementation of food policies and guidelines, socioeconomic status (SES) prevails as a significant driver of FVC across the world.

Low socioeconomic status (SES) is associated with low FVC, when compared to high SES households, in both developed and developing economies (12–20). Low SES households typically have a less diverse diet (21), and often consume a smaller variety of fruit and vegetable products (i.e., smaller number of unique items) compared to high SES (17, 22). Other measures of SES, including low educational attainment and low social class, have also been independently associated with lower fruit and vegetable variety (17). Low SES households consume less fruits and vegetables across the full spectrum of processing, including process-unspecified fruit and vegetables (15, 22–31), fresh fruit and vegetables (32), process-unspecified vegetables (33–40), fresh or frozen vegetables (41–43), process-unspecified fruit (24, 34–36, 39, 41, 44–46), fresh fruit (37, 42, 47), and fruit juices (34, 37, 45). In most studies observed, SES is measured by individual/household income or other proximal socioeconomic measures such as occupation, educational attainment, wealth, or income-to-poverty ratio (IPR) (15, 33, 34, 48–51). Some studies have shown that food neophobia is inversely associated with education and income (52, 53), which may, in part, account for socioeconomic differences since low-income consumers are more reluctant to try new foods that contribute to dietary diversity.

Differences in consumption based on the fruit and vegetable processing methods (and degrees) have been associated with varying health outcomes (54, 55). For example, Oyebode et al. (54) observed protective properties from overall vegetables, fresh salad, and fresh fruit within the context of all-cause cancer and cardiovascular disease mortality. However, at the same time, it was observed that frozen and canned fruit consumption, but not vegetables, are associated with increased mortality that is possibly tied to sugar content (54). Other prospective studies have shown that consumption of a larger variety of fruits and vegetables reduced the risk of type-2 diabetes and some cancers, independent of the quantity of intake (56, 57). The nutritional content of a fruit or vegetable is dependent on a number of factors involved in their processing including time/duration, level of heat involved in cooking (e.g., blanching before freezing), amount of food additives like sugar or salt, or long-term storage conditions (i.e., frozen, refrigerated, or room temperature) (58, 59). Variations in the method of cooking, processing, and storing, compounded by individual differences in the fruit or vegetable, can influence the availability and overall content of nutritional components like vitamins (A, B, C, and E), minerals, fibers, carotenoids, and phenolic compounds (58, 59). The results of these studies highlight the importance of understanding FVC at a more granular level and how households switch between various forms of FVC (fresh, frozen, canned, dried), and whether SES plays a role.

Beyond SES and level of processing, FVC is dependent on factors that act across a number of pathways that vary in mechanism and scale, which contribute to the variations seen in consumption across the globe (4, 11). Many of these factors are, in fact, intimately woven with SES. For instance, sociodemographic and cultural factors influence FVC in both children and adults (12–14, 60). These factors include indicators like age and sex (12, 13, 61), as well as cultural background and race (62, 63). Familial structures are also found to moderate FVC in children and adolescents via living arrangements (e.g., one- vs. two- parent households) (12), parental modeling/intake (13, 64), and both family rules and parental encouragement (64). In adults, marital status is found to influence FVC, with married couples having higher FVC than singles (14).

Accessibility, in all its dimensions, also plays a significant role in mediating FVC. Accessibility—whether it be physical, financial, or mental—is a key factor influencing FVC across a number of direct and indirect pathways. For example, the physical accessibility of fruits and vegetables on a supermarket shelf is dependent on the functioning of an adequate and stable supply chain. The agri-food supply chain, in turn, operates as a complex system spanning from input of raw materials, to farming, processing, packaging, and distribution (65). Physical accessibility also extends to the point of market access, where factors like having car access or grocery/food retail outlets within the neighborhood, supermarket choice, and access to home-grown produce via a home or community garden influence FVC (14, 51, 66, 67). For adults, FVC can also be mediated by consumption of fruits and vegetables from home gardens or farms (66–69). Furthermore, children and adolescents with greater physical accessibility (i.e., having fruits and vegetables present at home or at school) report higher FVC (13, 68). In alignment with the reported differences in FVC linked to SES, financial accessibility also influences consumption pathways predominantly through the mechanism of price. FVC has been found to be inversely correlated with price (20, 70), with fruits having a higher price elasticity than vegetables (71). Low SES consumers are the most price-sensitive (72) and are more likely to travel beyond their nearest supermarket to access low-cost alternatives (51). Financial accessibility has been the target of fiscal policy instruments designed to increase FVC and food security (72) through mechanisms like taxation [e.g., sugar-sweetened beverages (73)], or subsidization of healthier foods [e.g., rebates for fruits and vegetables in the Supplemental Nutrition Assistance Program (70, 74)]. Lastly, mental accessibility refers to the extent to which an individual has the knowledge to access fruits and vegetables for consumption, which is largely influenced by the sociodemographic variables (12–14, 60) and prior dietary knowledge or food skills (13, 14, 70). Mental accessibility is the target of many nutrition education and marketing initiatives implemented to raise awareness, salience, and top-of-mind knowledge that ultimately drives consumption (75–77). Many public health initiatives (78), such as those adopted by over 100 countries to implement food-based dietary guidelines that inform national food, nutrition, and health policies and programs (8), also act on pathways to boost awareness and mental accessibility of fruits and vegetables over time.

FVC follows a seasonal pattern (55, 79–84) that can be traced to agri-food production systems and availability of non-processed fresh products (14). In rural and traditional contexts, seasonality can be more pronounced where food consumption is often tied closer to local food markets and agricultural production (85, 86). Food prices, employment status (especially for seasonal workers), and changes in dietary diversity have been identified as pathways by which seasons can affect household food security (87, 88). Fluctuations in FVC over time may be tied to negative health consequences via pathways associated to oxidative stress (55, 82). Seasonal differences by individual/variety of fruit and vegetable, or by level of processing, have not yet been studied in-depth.

## Context And Objectives

The majority of studies to-date have either grouped fruits and vegetables together or used inconsistent definitions for categorizing fruits and vegetables when investigating consumption or expenditure patterns (15, 22–47). This body of evidence largely relies on dietary questionnaires that are subject to bias (89–93), although some statistical methods can be used to correct for this (94, 95). Given the diversity and complexity of colors, flavors, textures, and nutritional qualities in fruits and vegetables, compounded by the effects of marketing and messaging tying fruits and vegetables products together, a deeper understanding of the unique properties, drivers, and consumption patterns for fruits and vegetables individually is warranted (96). Furthermore, vegetables have been associated with more protective factors against cancers and cardiovascular diseases in comparison to fruits (54). The present investigation segregates vegetables from fruits and dissects vegetable expenditure patterns to contribute empirical evidence on the interplay between drivers of expenditure as a proxy for consumption, as well as tease apart the complex relationships by which SES acts as a driver over time and across food processing levels.

The present study is designed with the Canadian food guide in mind, which is different from other countries. We examine retrospective consumer loyalty program data obtained from one of the leading Canadian grocery retailers. The dataset provides a unique testbed to dissect vegetable expenditure patterns and socioeconomic inequity pathways. Vegetable expenditure is tracked using total expenditure as a consistent metric to account for vegetables of different varieties and forms. Using the unique identifier assigned by the retailer's consumer loyalty program, individual food purchases were analyzed over a 32-month time period from 2015 to 2017. Individual purchasing data was linked via postal code to sociodemographic indicators obtained from the 2016 Canadian census. Together, the linked sociodemographic and consumer loyalty program data enabled a series of analyses aimed at dissecting vegetable expenditure as a function of SES.

## Materials and Methods

### Data Source and Sample Inclusions

The majority of food retailers, like grocery stores, capture all sales transactions electronically. The sales data are used for store planning and sales forecasting (97), as well as conducting analytics to understand changing consumer needs (98, 99). Many retailers also offer voluntary loyalty programs for consumers to join, usually offering consumers rewards (e.g., member-only discounts, coupons, or cash-back) in return. This allows retailers to better track purchase patterns at the individual household level (98, 99), as well as build value perception and brand loyalty (100).

Data was obtained for all retail transactions made by loyalty program members at grocery stores in Quebec, Canada from February 1st, 2015 to September 30th, 2017. All member purchases can be traced, so this information represents typical panel data. Members provided postal codes of their residence when they signed up for the loyalty program, although we do not observe any other member-specific demographics. Although we cannot disclose the exact total number of members to protect the retailer's identity, a little <300,000 are frequent consumers. We selected only frequent consumers, defined as those who shopped at least once per month during the entire data duration, to ensure the data from this retailer can reflect majority of the member's food purchases. Infrequent consumers may visit other grocery stores, and the data are not representative of their true shopping baskets. Extreme outliers, defined as frequent consumers with an average monthly food basket >99.9 percentile, and consumers without postal code-level income were removed from the sample.

### Measures

#### Consumer Food Expenditure Share on Vegetables

Following previous methods used for assessing the healthfulness of food purchase from agricultural economics (101, 102), food expenditure share allocated to vegetables was selected as the dependent variable in the analyses. All vegetable Universal Product Codes (UPCs) sold by the grocery retailer were classified using a framework that was informed by a review of vegetable processing techniques (103–105) in relation to the vegetable retail environment of the grocery retail partner. Independent coders classified all vegetable products into one of five groups by level of processing, which included non-processed fresh vegetables, fresh cut vegetables, fresh prepared vegetables, canned vegetables, and frozen vegetables. The non-processed fresh vegetable category included whole, non-cut, and non-processed vegetables and fresh herbs. The fresh cut vegetable category included cut vegetables, with or without dip. The fresh prepared vegetable category included all types of salad (e.g., store-prepared salads or manufacturer-prepared salads), as well as appetizers or small plates prepared with vegetables. The canned vegetable category included all canned vegetable products, while the frozen vegetable category included all frozen vegetable products. Note that main courses (e.g., prepared or frozen vegetarian entrées), dehydrated vegetables, and fermented/pickled vegetables were excluded from these analyses.

Vegetable expenditure share of shopping baskets were constructed for consumer *i* and vegetable group *g* in month *m* as:

(1) $$Food\;expenditure\;share\;of\;vegetable\;types_{igm}=\frac{vegetable\;expenditure_{igm}}{total\;food\;expenditure_{im}}$$

We also calculate the total vegetable share of food expenditure by

(2) $$Food\;expenditure\;share\;of\;all\;vegetables_{im}=\frac{total\;vegetable\;expenditure_{im}}{total\;food\;expenditure_{im}}$$

The sum of expenditure shares (Equation 1) over all the groups equals the total vegetable food expenditure (Equation 2). By introducing the two levels of measures, we can investigate the impact of seasonality and SES on the overall vegetable expenditure, as well as by groups, to gain more insights on consumer expenditure patterns.

#### Postal Code-Level Neighborhood Census Characteristics and Store-Level Vegetable Variety

Data from the loyalty program data only contained the home postal code for each consumer, but no individual demographic information. Therefore, we used neighborhood socioeconomic and household characteristics as proxy for individual socioeconomic and household characteristics. We obtained the 2016 Canadian Census data and matched each consumer to a dissemination area (DA) by postal code. A DA is the smallest standard geographic area for which all census data are disseminated, with an average population of 400 to 700 persons. We included the following neighborhood socioeconomic and household characteristics: (1) Population density; (2) Proportion of census families with at least one child; (3) Proportion of the population aged 15 years and over that were not married (Never married, Separated, Divorced, or Widowed); (4) Proportion of single-parent families; (5) Median family income; (6) Proportion of the population aged 15 years and over that were employed; and (7) Proportion of the population aged 15 years and over with post-secondary education. To control for in-store variety, we included the number of fruit and vegetable UPCs in each store as a control variable in the model.

### Analytical Approach

Our goal is to understand the relationship among monthly food expenditure share of vegetables, seasonality, consumer socioeconomic and household characteristics, and store-level vegetable variety. We used a panel random effect model to control for unobserved differences among individual households (106, 107). In addition, not all consumers purchased all vegetable groups every month. Only non-processed fresh vegetables are purchased almost every month. We frequently observed zero purchases in the frozen, canned, fresh-cut, and fresh prepared vegetable categories. This is a typical censored data problem, and we employed a tobit model to deal with the censored nature of the dependent variables (108, 109). Finally, we used clustered standard errors with clusters of consumers to account for the non-independence of repeated measures within individuals (110). We estimated a separate random effect tobit model separately for the total vegetable expenditure and each of the vegetable groupings. Positive β coefficients represent an increase in vegetable expenditure.

To better evaluate the impact of socioeconomical variables on seasonal consumption patterns, we also conducted a stratified analysis by (median) splitting the sample into low- and high- family income subgroups. We then estimated the same model separately for the low- and high-income subgroups. This analysis helps to understand how the influence of postal code-level neighborhood census characteristics and store characteristics act differently between two subgroups. All analyses were carried out with STATA, version 13 (Stata, College Station, TX, USA). Confidence intervals (CI) for hypothesis tests were constructed at the 95% confidence level.

## Results

### Descriptive Results

Table 1 reports the characteristics of consumers that were included in the sample. On average, consumers spent CAD 286.96 per month on food items with large variation (SD = CAD 212.03). Consumers spent CAD 24.26 per month on vegetables on average, accounting for 8.35% of total food expenditure with a large variation (SD = 6.29%). The observed large variations show the importance of analyzing data at the individual-level compared to store-level sales.

**Table 1:** Characteristics of consumers and grocery retail stores in Quebec, Canada.

	**Mean**	**SD**	**Minimum**	**25th percentile**	**50th percentile**	**75th percentile**	**Maximum**
**Consumer expenditure characteristics**							
Monthly food expenditure ($)	286.96	212.03	0.03	129.87	234.95	390.76	1415.58
Monthly vegetables expenditure ($)	24.26	24.51	0.00	6.77	17.04	34.08	877.04
Average monthly food expenditure share on vegetables (%)	8.35	6.29	0.00	4.07	7.46	11.41	100.00
**Food expenditure share for groups of vegetables, by processing level**							
Non-processed fresh (%)	6.88	5.69	0.00	2.96	5.96	9.56	100.00
Fresh cut (%)	0.17	0.77	0.00	0.00	0.00	0.00	100.00
Fresh prepared (%)	0.50	1.43	0.00	0.00	0.00	0.00	100.00
Canned (%)	0.56	1.24	0.00	0.00	0.00	0.75	100.00
Frozen (%)	0.24	1.02	0.00	0.00	0.00	0.00	100.00
**Postal code-level neighborhood census characteristics**							
Population density (/square meter)	0.003	0.005					
Proportion of census families with at least one child	44.95	12.98					
Proportion of the population aged 15 years and over were not married (including never married, separated, divorced, or widowed)	42.11	10.80					
Proportion of single-parent families	16.19	7.45					
Median family income (/$1,000)	67.31	27.09					
Proportion of the population aged 15 years and over with post-secondary education	59.05	11.83					
Proportion of the population aged 15 years and over were employed	58.71	11.70					
**Store characteristics**							
Number of fruit and vegetable UPCs (/1,000)	1.89	0.24					

*SD, Standard Deviation; UPC, Universal Product Code*.

Table 1 also provides further breakdown of vegetables into five groups. The largest group, non-processed fresh vegetables, accounted for 6.88% of monthly food expenditure. The spending on this vegetable group is more than 10 times larger than on any other group. Vegetables of higher processing levels (i.e., fresh cut, fresh prepared, frozen, and canned) were consumed far less frequently and, on average, collectively contributed to only 1.47% of the overall food expenditure. Canned vegetables and fresh prepared vegetable groups each contributed to around 0.5% of the monthly food expenditure. The remaining two groups, frozen vegetables, and fresh cut vegetables, each contributed 0.25% or less of the monthly food expenditure. The percentile measures (25, 50, 75%) show that non-processed fresh vegetables are purchased in majority of the monthly observations, whereas fresh cut, fresh prepared, and frozen vegetables are purchased in <25% of the monthly observations. Consumers obviously consider processed vegetables as very different products, and only non-processed fresh vegetables are universally accepted and purchased almost every month. Furthermore, Table 1 provides the summary statistics of postal code-level neighborhood census characteristics, including income, educational attainment, employment, and other variables. Finally, the store characteristics include a count of distinct fruit and vegetable UPCs to indicate the variety of products available for consumers to choose from in a given store in a given month. For example, different varieties of lettuce (Romaine vs. Boston) or different packaging of lettuce are treated as distinct products. A typical store can carry close to two thousand distinct UPCs for fruits and vegetables with vast variations over time and across different stores.

**Figure 1:**
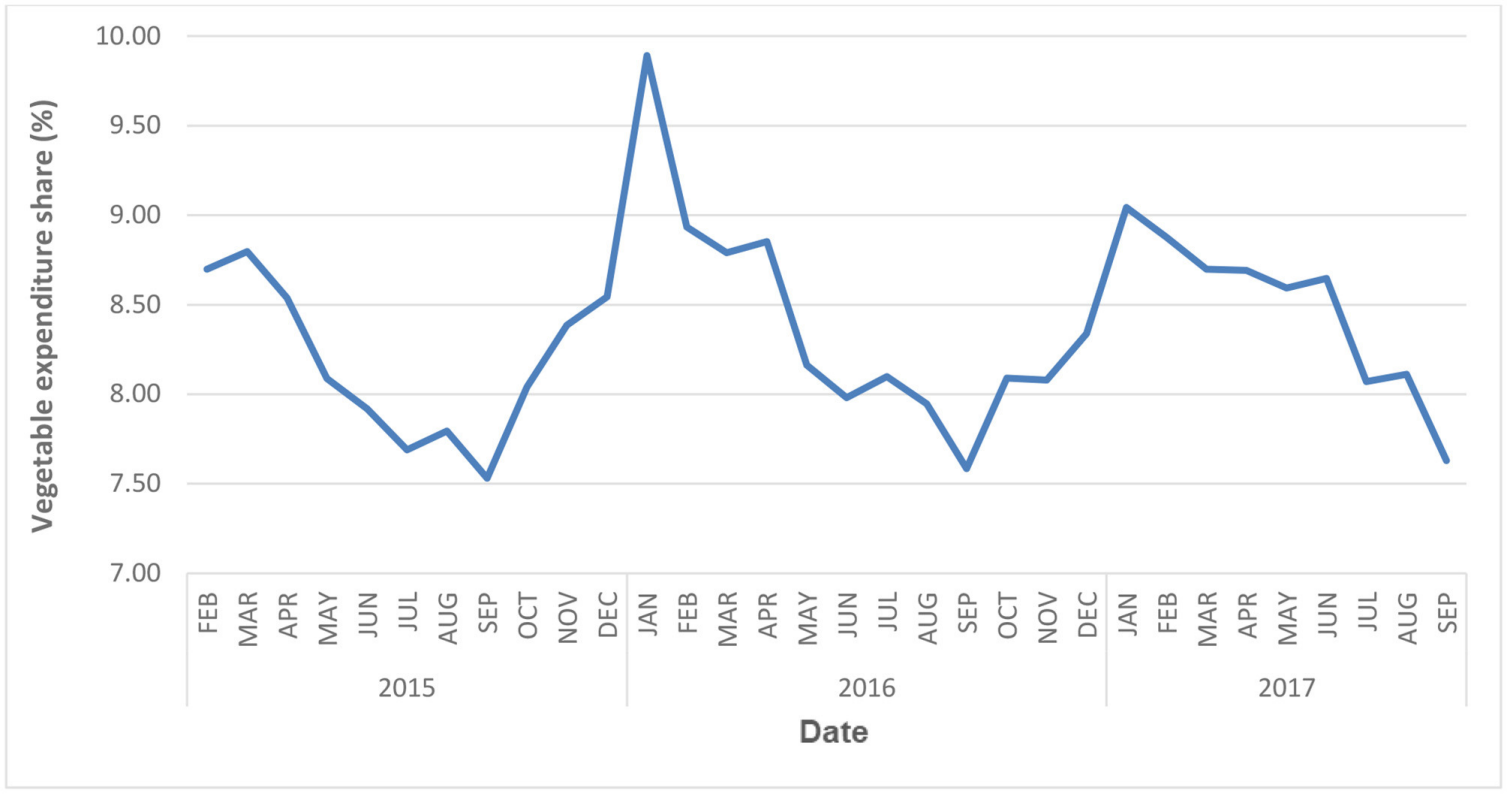
Average vegetable expenditure share, month over month.

Figure 1 plots the share of vegetables in the food basket over time. The plot shows a strong seasonal pattern over the duration of the study. In general, the food expenditure share of vegetables peaks in winter, then declines to its lowest point in fall before it starts rising again. The highest recorded value of vegetable expenditure share is 9.89% in January 2016 and the lowest value is 7.53% in September 2015 throughout the study period.

**Figure 2:**
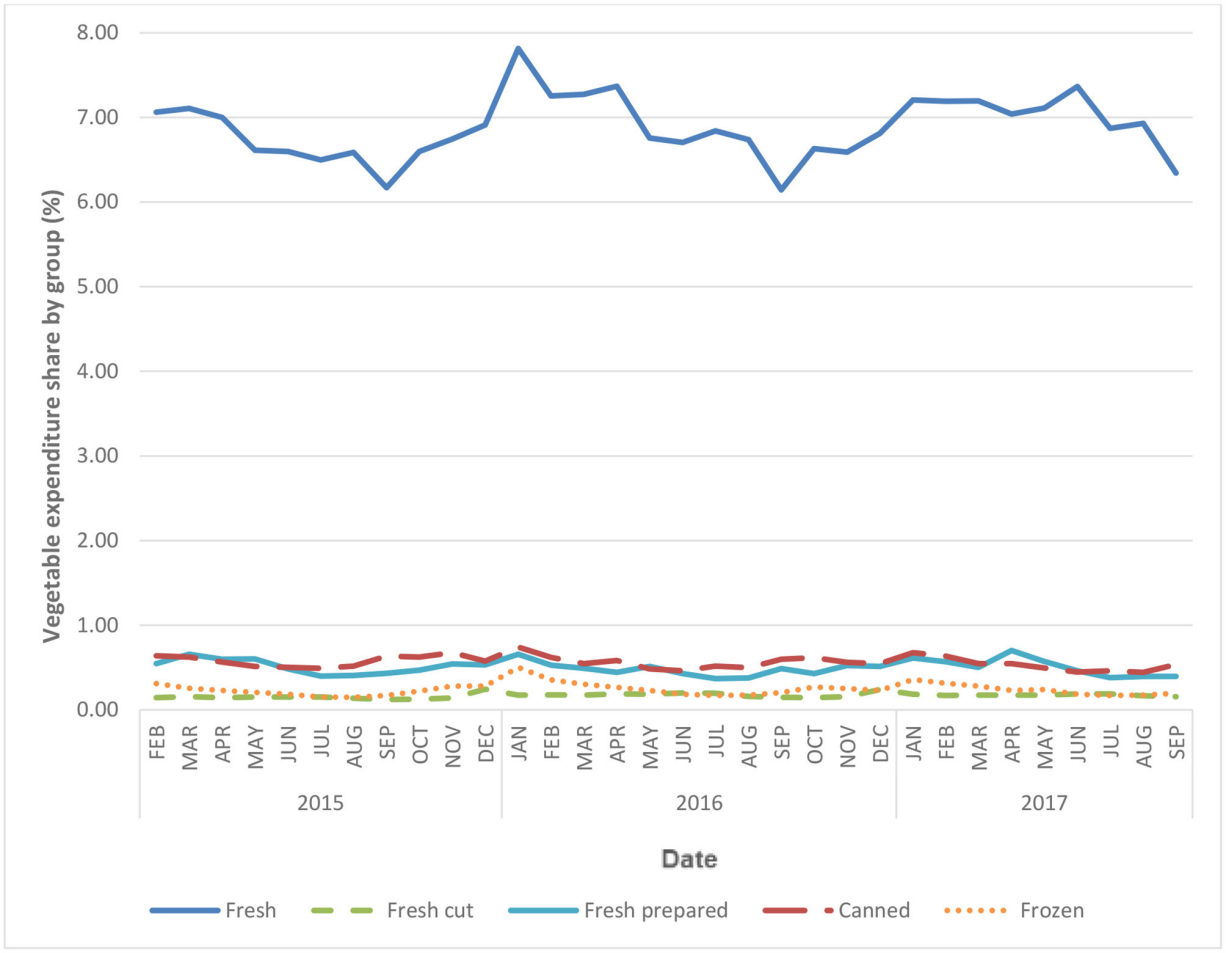
Average vegetable expenditure share by food group, month over month. The definition for each subheading: (1) non-processed fresh: including whole, non-cut, and non-processed vegetables and fresh herbs; (2) fresh cut: including cut vegetables, with or without dip; (3) fresh prepared: including all types of salad (e.g., store-prepared salads or manufacturer-prepared salads), as well as appetizers or small plates prepared with vegetables; (4) canned vegetable: including all canned vegetable products; (5) frozen vegetable: including all frozen vegetable products.

Figure 2 plots the shares of vegetable groups across processing levels. It is very clear that varying patterns emerge for the varying groups of vegetables. Regardless of the processing level, all vegetable groups illustrated seasonal variation with the highest share of overall food expenditure in winter and the lowest in summer. There are some differences in the patterns though. Fresh cut vegetables demonstrated the least seasonal variance when compared to other groups, whereas frozen vegetables seem to produce the largest spikes over time. Canned and fresh prepared products are roughly equal in their expenditure share values and seasonal movements.

### Analytical Results—Overall Sample

We first estimated the expenditure model with all consumers to examine the overall seasonality and the influence of demographics and store-level food variety on vegetable expenditure shares. Table 2 reports the estimation results for overall vegetables and each group according to their level of processing.

**Table 2:** Results of the association between postal code-level neighborhood census characteristics, store characteristics, and vegetable expenditure share by group (using *food basket as denominator*) in Quebec, Canada.

	Overall^*^			Non-processed fresh^*^			Fresh cut^*^			Fresh prepared^*^			Canned^*^			Frozen^*^			
	**β**	**Robust SE**	***P* > *z***	**β**	**Robust SE**	***P* > *z***	**β**	**Robust SE**	***P* > *z***	**β**	**Robust SE**	***P* > *z***	**β**	**Robust SE**	***P* > *z***	**β**	**Robust SE**	***P* > *z***	*p*-value^*^
**Year**																			
2015	Ref	Ref	Ref	Ref	Ref	Ref	Ref	Ref	Ref	Ref	Ref	Ref	Ref	Ref	Ref	Ref	Ref	Ref	
2016	0.12	0.005	<0.001	0.15	0.004	<0.001	0.05	0.004	0	−0.22	0.005	<0.001	−0.06	0.002	<0.001	0.17	0.01	<0.001	
2017	0.17	0.01	<0.001	0.21	0.01	<0.001	0.01	0.005	0.053	−0.07	0.01	<0.001	−0.13	0.003	<0.001	0.02	0.01	0.001	
**Month**																			
January	Ref	Ref	Ref	Ref	Ref	Ref	Ref	Ref	Ref	Ref	Ref	Ref	Ref	Ref	Ref	Ref	Ref	Ref	
February	−0.59	0.01	<0.001	−0.29	0.01	<0.001	−0.15	0.01	<0.001	−0.60	0.01	<0.001	−0.203	0.004	<0.001	−0.68	0.01	<0.001	<0.001
March	−0.63	0.01	<0.001	−0.24	0.01	<0.001	−0.06	0.01	<0.001	−0.53	0.01	<0.001	−0.386	0.004	<0.001	−0.96	0.01	<0.001	<0.001
April	−0.70	0.01	<0.001	−0.30	0.01	<0.001	−0.08	0.01	<0.001	−0.46	0.01	<0.001	−0.371	0.004	<0.001	−1.31	0.01	<0.001	<0.001
May	−1.09	0.01	<0.001	−0.58	0.01	<0.001	−0.14	0.01	<0.001	−0.55	0.01	<0.001	−0.628	0.005	<0.001	−1.48	0.01	<0.001	<0.001
June	−1.20	0.01	<0.001	−0.53	0.01	<0.001	−0.09	0.01	<0.001	−1.08	0.01	<0.001	−0.713	0.005	<0.001	−1.92	0.01	<0.001	<0.001
July	−1.45	0.01	<0.001	−0.70	0.01	<0.001	−0.07	0.01	<0.001	−1.49	0.01	<0.001	−0.675	0.005	<0.001	−2.15	0.01	<0.001	<0.001
August	−1.46	0.01	<0.001	−0.69	0.01	<0.001	−0.21	0.01	<0.001	−1.42	0.01	<0.001	−0.655	0.005	<0.001	−2.12	0.01	<0.001	<0.001
September	−1.83	0.01	<0.001	−1.23	0.01	<0.001	−0.34	0.01	<0.001	−1.26	0.01	<0.001	−0.326	0.004	<0.001	−1.75	0.01	<0.001	<0.001
October	−1.28	0.01	<0.001	−0.76	0.01	<0.001	−0.43	0.01	<0.001	−0.92	0.01	<0.001	−0.278	0.005	<0.001	−1.20	0.01	<0.001	<0.001
November	−1.13	0.01	<0.001	−0.72	0.01	<0.001	−0.15	0.01	<0.001	−0.63	0.01	<0.001	−0.281	0.005	<0.001	−1.09	0.01	<0.001	<0.001
December	−0.87	0.01	<0.001	−0.49	0.01	<0.001	0.08	0.01	<0.001	−0.57	0.01	<0.001	−0.448	0.005	<0.001	−1.20	0.01	<0.001	<0.001
**Postal code-level neighborhood census characteristics**																			
Monthly food expenditure (log transformation)	−0.35	0.01	<0.001	−0.31	0.01	<0.001	1.06	0.01	<0.001	1.34	0.005	<0.001	0.69	0.004	<0.001	1.23	0.006	<0.001	
Population density (/square meter)	−21.67	2.19	<0.001	−29.14	2.01	<0.001	−2.83	1.17	0.02	8.25	1.43	<0.001	4.89	0.69	<0.001	27.53	2.021	<0.001	
Proportion of census families with at least one child	−0.01	0.001	<0.001	−0.01	0.001	<0.001	0.003	0.0005	<0.001	−0.005	0.001	<0.001	−0.001	0.0003	<0.001	0.01	0.001	<0.001	
Proportion of the population aged 15 years and over were not married	0.01	0.001	<0.001	0.002	0.001	0.05	−0.00002	0.001	0.97	−0.002	0.001	0.07	0.004	0.0004	<0.001	0.02	0.001	<0.001	
Proportion of single-parent families	−0.01	0.002	<0.001	−0.01	0.001	<0.001	0.001	0.001	0.20	0.01	0.001	<0.001	−0.0004	0.0005	0.45	−0.01	0.001	<0.001	
Median family income (/$1,000)	0.01	0.001	<0.001	0.01	0.0005	<0.001	0.003	0.0002	<0.001	0.01	0.0003	<0.001	−0.001	0.0001	<0.001	−0.001	0.0004	0.08	
Proportion of the population aged 15 years and over with post-secondary education	0.03	0.001	<0.001	0.02	0.001	<0.001	−0.004	0.0005	<0.001	0.01	0.001	<0.001	0.0004	0.0003	0.15	0.01	0.001	<0.001	
Proportion of the population aged 15 years and over were employed	−0.01	0.001	<0.001	−0.01	0.001	<0.001	0.01	0.0005	<0.001	0.01	0.001	<0.001	0.0004	0.0003	0.10	−0.01	0.001	<0.001	
**Store characteristics**																			
Number of fruit and vegetable UPCs (/1,000)	1.07	0.03	<0.001	0.96	0.03	<0.001	−0.004	0.02	0.82	0.45	0.02	<0.001	−0.09	0.01	<0.001	−0.05	0.03	0.13	
_cons	7.89	0.10	<0.001	6.47	0.09	<0.001	−9.41	0.07	<0.001	−11.85	0.07	<0.001	−3.78	0.04	<0.001	−11.65	0.09	<0.001	
sigma_u	3.72			3.39															
sigma_e	5.01			4.53															
rho	0.36			0.36															

*SE, Standard Error; UPC, Universal Product Code; Number of observations = 9,077,691*.

* *The definition for each subheading: (1) non-processed fresh: including whole, non-cut and non-processed vegetables and fresh herbs; (2) fresh cut: including cut vegetables, with or without dip; (3) fresh prepared: including all types of salad (e.g., store-prepared salads or manufacturer-prepared salads), as well as appetizers or small plates prepared with vegetables; (4) canned vegetable: including all canned vegetable products; (5) frozen vegetable: including all frozen vegetable products*.

The overall share of vegetables in the food basket increased by 0.17% (*p* < 0.001) from 2015 to 2017. However, not all vegetable groups showed a uniform increase. Non-processed fresh vegetables were the core driver of the observed increase, rising by 0.21% (*p* < 0.001) over the two-year period. Fresh cut and frozen vegetable expenditures also increased, however, fresh prepared and canned products decreased by 0.07 and 0.13% (*p* < 0.001) respectively.

The degree of seasonal variation across the five groups by processing level are significantly different from one another (*p* < 0.001), suggesting each group of vegetables is characterized by its own unique pattern of expenditure. Taking January as the reference month, significant seasonal variations are observed from 1 month to another across all the vegetable processing levels studied (*p* < 0.001). Vegetable expenditure share was the highest in winter, and the lowest in late summer, both overall and across groupings by level of processing. Frozen vegetables demonstrated the greatest seasonal variation with a 2.15% decrease in expenditure share between January and July (β = −2.15, *p* < 0.001). This confirms the visual observations in Figure 2. Fresh prepared vegetable expenditure shares also decreased by 1.49% (*p* < 0.001) from January to July, while non-processed fresh vegetables decreased by 1.23% (*p* < 0.001) between January and September. Fresh cut and canned vegetable groups demonstrated significant seasonal variations of <1 percent.

Demographic characteristics had heterogeneous effects on vegetable expenditure share. Population density was the strongest driver of vegetable expenditure share (β = −21.67, *p* < 0.001). However, its effects differed across the vegetable processing levels. High population density had strong positive effects on fresh prepared, canned, and frozen vegetables, but negative effects on non-processed fresh products (*p* < 0.001). In terms of household familial relationships, having children increased expenditure shares of fresh cut and frozen vegetables, but decreased shares of all others (*p* < 0.001). In their food basket, single-parent families spent less on non-processed fresh, fresh prepared, and frozen vegetables (*p* < 0.001). In addition to demographics, SES played a significant role that influenced the share of wallet devoted to vegetables.

Heterogeneous effects were again observed when evaluating the three variables used as measures for SES: income, educational attainment, and overall food basket expenditure. Low family income had negative effects on all fresh vegetables, but positive effects on canned vegetables. Low educational attainment decreased the shares of all product groups, except fresh cut vegetables. Low employment increased shares of non-processed fresh and frozen vegetables but decreased the shares of fresh cut and fresh prepared vegetables. In addition, consumers who spent less on their overall food basket were more likely to spend on non-processed fresh vegetables, and less likely to purchase fresh cut, fresh prepared, canned, and frozen vegetables as part of their food basket. These findings demonstrate that SES factors can also have a strong influence on the vegetable purchases, and our findings on their heterogeneous effects are new.

In the grocery retail environment, greater store-level variety of fruits and vegetable UPCs promoted expenditure on non-processed fresh and fresh prepared vegetables but reduced that of canned vegetables. Table 3 provides a summary of the directions of all variables.

**Table 3:** Summary of the association between postal code-level neighborhood census characteristics and vegetable expenditure share by food group (using *food basket as denominator*).

	**Overall**	Non-processed fresh^*^	Fresh cut^*^	Fresh prepared^*^	Canned^*^	Frozen^*^
**Postal code-level neighborhood census characteristics**						
Monthly food expenditure (log transformation)	**–**	**–**	**+**	**+**	**+**	**+**
Population density (/square meter)	**–**	**–**	**–**	**+**	**+**	**+**
Proportion of census families with at least one child	**–**	**–**	**+**	**–**	**–**	**+**
Proportion of the population aged 15 years and over were not married	**+**	**+**	.	.	**+**	**+**
Proportion of single–parent families	**–**	**–**	.	**+**	.	**–**
Median family income (/$1,000)	**+**	**+**	**+**	**+**	**–**	.
Proportion of the population aged 15 years and over with post-secondary education	**+**	**+**	**–**	**+**	.	**+**
Proportion of the population aged 15 years and over were employed	**–**	**–**	**+**	**+**	.	**–**
**Store characteristics**						
Number of fruit and vegetable UPCs (/1,000)	**+**	**+**	.	**+**	**–**	.

*UPC, Universal Product Code; +, significantly positive association; -, significantly negative association; ., no association; Number of observations = 9,077,691*.

* *The definition for each subheading: (1) non-processed fresh: including whole, non-cut and non-processed vegetables and fresh herbs; (2) fresh cut: including cut vegetables, with or without dip; (3) fresh prepared: including all types of salad (e.g., store-prepared salads or manufacturer-prepared salads), as well as appetizers or small plates prepared with vegetables; (4) canned vegetable: including all canned vegetable products; (5) frozen vegetable: including all frozen vegetable products*.

The results presented above highlight the overall significance that several drivers of consumer vegetable expenditure play and provides evidence of significant seasonality across processing levels. Great variability was observed for overall food and vegetable expenditures in the overall sample; therefore, further investigation was warranted. It has been previously observed that a significant stratification may be observed across socioeconomic levels. Low SES, among other factors, can be predictive of lower than adequate purchase/intake of fruits and vegetables (17, 18, 20, 111). Other studies have shown that both low income and low educational attainment have similar effects on food consumption, and have been associated with lower consumption (15, 22, 34, 112, 113) and reduced purchasing (41, 114, 115) of vegetables compared to high-income counterparts. Therefore, to further dissect the consumer expenditure patterns as a function of SES, the consumer population was divided into two groups, low- vs. high-income, and analyses were repeated using the same methodology as for the whole sample. The results of the stratified analysis are presented below, still encompassing a large range of variability within each income subgroup.

### Analytical Results – Stratified Sample by Low vs. High Income Subgroups

The results of the stratified analysis by low- and high- income are presented in Table 4. Descriptive statistics for the stratified sample are reported in Supplementary Table 1 and illustrated in Supplementary Figure 1.

**Table 4:** Results of the association between postal code-level neighborhood census characteristics, store characteristics, and vegetable expenditure share, stratified by low-, and high- income (using *food basket as denominator*).

	**Low**			**High**		
	**β**	**Robust SE**	***P* > *z***	**β**	**Robust SE**	***P* > *z***
**Year**						
**2015**	**(Reference)**			**(Reference)**		
2016	0.14	0.01	<0.001	0.10	0.01	<0.001
2017	0.19	0.01	<0.001	0.15	0.01	<0.001
**Month**						
**January**	**(Reference)**			**(Reference)**		
February	−0.50	0.01	<0.001	−0.68	0.01	<0.001
March	−0.54	0.01	<0.001	−0.73	0.01	<0.001
April	−0.58	0.01	<0.001	−0.82	0.01	<0.001
May	−0.90	0.01	<0.001	−1.27	0.01	<0.001
June	−1.00	0.01	<0.001	−1.41	0.01	<0.001
July	−1.19	0.01	<0.001	−1.71	0.01	<0.001
August	−1.14	0.01	<0.001	−1.77	0.01	<0.001
September	−1.63	0.01	<0.001	−2.03	0.01	<0.001
October	−1.21	0.01	<0.001	−1.36	0.01	<0.001
November	−1.09	0.01	<0.001	−1.17	0.01	<0.001
December	−0.80	0.01	<0.001	−0.95	0.01	<0.001
**Postal code-level neighborhood census characteristics**						
Monthly food expenditure (log transformation)	−0.33	0.01	<0.001	−0.38	0.01	<0.001
Population density (/square meter)	−16.81	2.48	<0.001	−32.18	5.34	<0.001
Proportion of census families with at least one child	−0.007	0.001	<0.001	−0.02	0.001	<0.001
Proportion of the population aged 15 years and over were not married	−0.002	0.002	0.28	0.01	0.003	<0.001
Proportion of single-parent families	−0.01	0.002	<0.001	−0.008	0.003	0.01
Median family income (/$1,000)	0.001	0.002	0.72	0.01	0.001	<0.001
Proportion of the population aged 15 years and over with post-secondary education	0.02	0.001	<0.001	0.04	0.001	<0.001
Proportion of the population aged 15 years and over were employed	−0.007	0.001	<0.001	−0.001	0.001	0.60
**Store characteristics**						
Number of fruit and vegetable UPCs (/1,000)	0.83	0.05	<0.001	1.55	0.05	<0.001
_cons	9.00	0.16	<0.001	6.16	0.16	<0.001
sigma_u	3.78			3.66		
sigma_e	4.98			5.05		
rho	0.37			0.34		

*SE, Standard Error; UPC, Universal Product Code; Number of observations (low group) = 4,549,180; Number of observations (high group) = 4,528,511*.

Significant seasonal variation was observed month over month across all vegetable processing levels (p <0.001) for both low- and high-income groups. Both groups followed the same trend, with vegetable expenditure highest in winter (January) and lowest in late summer (September), with observed expenditure share decreases from peak to valley of 1.63% (*p* < 0.001) for low-income consumers and 2.03% (*p* < 0.001) for high-income consumers. This provides an evidence that high-income consumers exhibit the greatest magnitude of spending variations over time.

Consumers in the low-income group, on average, spent CAD $270.61 (SD = $200.25) on food and allocated 8.12% (SD = 6.28%) of their food basket to vegetables, with large variability observed within the subgroup. The subgroup also increased their vegetable expenditure by 0.19% (*p* < 0.001) from 2015 to 2017. Low-income consumers devoted the greatest share of wallet to non-processed fresh vegetables (6.68%) and the least share to fresh cut (0.15%). Frozen vegetables were allocated 0.26%, and canned 0.58%, by consumers in the low-income group on average.

Expenditure share devoted to vegetables increased by 0.15% (*p* < 0.001) over the study period for consumers in the high-income group, who on average spent CAD $303.39 (SD = $222.03) on food each month. In contrast to the low-income group, consumers in the high-income group allocated a significantly greater (*p* < 0.001) share of wallet to fresh vegetables (i.e., non-processed fresh, fresh cut, and fresh processed), and a significantly smaller (*p* < 0.001) share to processed packaged vegetables (canned and frozen). The greatest difference was observed for non-processed fresh, where the high-income group allocated 7.07% and the low-income group allocated 6.68%.

Heterogeneous effects were observed when comparing drivers of vegetable expenditure for low- and high-income groups. High population density, households with children, and single-parent families were negative drivers of vegetable expenditure share for both low- and high- income groups. Population density had the strongest effect on high-income consumers (β = −32.18, p <0.001) when compared to low-income consumers (β = −16.81, *p* < 0.001). Tied to SES, income level only had a significant impact on the high-income group (β = 0.01, *p* < 0.001), while higher educational attainment was a positive driver of vegetable expenditure for both groups. The proportion of the population employed reduced the share of vegetables expenditures for the low-income group (β = −0.007, *p* < 0.001). The summary of the stratified analysis is presented with the directional findings in Table 5.

**Table 5:** Summary of the association between postal code-level neighborhood census characteristics and vegetable expenditure share by food group, stratified by low-, and high- income (using *food basket as denominator*).

			**Low**					**High**		
	Non-processed fresh^*^	Fresh cut^*^	Fresh prepared^*^	Canned^*^	Frozen^*^	Non-processed fresh^*^	Fresh cut^*^	Fresh prepared^*^	Canned^*^	Frozen^*^
**Postal code-level neighborhood census characteristics**					
Monthly food expenditure (log transformation)	**–**	**+**	**+**	**+**	**+**	**–**	**+**	**+**	**+**	**+**
Population density (/square meter)	**–**	.	**+**	**+**	**+**	**–**	.	**+**	**+**	**+**
Proportion of census families with at least one child	**–**	**+**	**–**	**–**	**+**	**–**	**+**	**–**	**–**	**+**
Proportion of the population aged 15 years and over were not married	**–**	**+**	**+**	**+**	**+**	**+**	**–**	.	.	**+**
Proportion of single–parent families	**–**	.	**+**	.	**–**	**–**	**+**	**+**	.	**–**
Median family income (/$1,000)	.	.	**+**	.	.	**+**	**+**	**+**	**–**	.
Proportion of the population aged 15 years and over with post-secondary education	**+**	**–**	**+**	**+**	**+**	**+**	**–**	**+**	**–**	**+**
Proportion of the population aged 15 years and over were employed	**–**	**+**	**+**	**–**	**–**	**–**	**+**	**+**	.	**–**
**Store characteristics**					
Number of fruit and vegetable UPCs (/1,000)	**+**	**–**	**+**	**–**	**–**	**+**	.	**+**	**–**	**+**

*UPC, Universal Product Code; +, significantly positive association; -, significantly negative association; ., no association; Number of observations (low group) = 4,549,180; Number of observations (high group) = 4,528,511*.

* *The definition for each subheading: (1) non-processed fresh: including whole, non-cut and non-processed vegetables and fresh herbs; (2) fresh cut: including cut vegetables, with or without dip; (3) fresh prepared: including all types of salad (e.g., store-prepared salads or manufacturer-prepared salads), as well as appetizers or small plates prepared with vegetables; (4) canned vegetable: including all canned vegetable products; (5) frozen vegetable: including all frozen vegetable products*.

Although the directions of majority of the sociodemographic variables are consistent between the two income groups, significant differences were observed for marriage status and educational attainment. As the proportion of the not married/single population aged 15 years and over increases for low-income consumers, the expenditure share of non-processed fresh vegetables decreases while the proportion of fresh cut vegetables increases. The opposite is observed for the high-income group; as the proportion of single households increase, the more they spend on non-processed fresh vegetables, and less on fresh cut. Consumers with higher educational attainment (i.e., those with post-secondary education) in the low-income group were more likely to allocate expenditure share to canned foods, whereas higher educated consumers in the high-income group were less likely to allocate funds to canned vegetables. In terms of store characteristics, an increase in the variety of fruit and vegetable UPCs has a significantly negative association with frozen vegetables for low-income consumers, but a significantly positive association for high-income consumers.

## Discussion

We used large-scale Canadian transactional retail data to study vegetable expenditure patterns at the store and consumer levels over a lengthy duration and a large geographic scope (116, 117). This approach addressed some disadvantages of the diet questionnaires approach used in previous studies to assess seasonal variation in vegetable consumption and prices (89–91). Self-reported dietary intake is subject to reporting error and bias and may be substantially inaccurate for calculation and evaluation (92, 93). Evidence also suggests that subjects do not easily distinguish between their current vs. usual intake, and that reporting bias shifts values toward intake during the season and time that the questionnaire is administered (118). Some researchers have, however, proposed statistical methods to correct for this error (94, 95). Recent survey data from Statistics Canada indicated that consumers in Quebec allocate 11.36% of their average monthly food expenditure (CAD $482.67) to vegetables (119); whereas we observed that 8.35% was allocated to vegetables from an average monthly food basket of CAD $286.96. This discrepancy suggests that vegetable expenditure may be over-reported by survey data; however, consumers could be augmenting their vegetable purchases at retail outlets outside of the grocery retail chain that we have partnered with for this study.

Overall, we observed a significant increase in vegetable expenditure share by 0.17% (*p* < 0.001) over the 3-year period. This finding suggests that the increase in vegetable expenditure share could be an artifact of increasing age, which is consistent with evidence that repeated exposure to unfamiliar vegetables can lead to increased liking for those vegetables over time (120, 121). Our study also revealed significant seasonal fluctuation for vegetable expenditure and that the fluctuation varies by their processing level. Our data showed the average expenditure share of vegetables reached the lowest share of 7.5% in late summer and the highest share of 9.9% in winter. The peak represented an approximate 50% increase compared to the valley. Also, this significant seasonal fluctuation is the main reason for the large standard deviations of food expenditure share. In addition, the patterns were similar between high income and low-income groups. Previous studies have shown that costs are one of the major factors preventing people from eating more fruits and vegetables (122). Therefore, it would be extremely challenging for low-income households to allocate 50% more budget to cover vegetables expenditure and maintain a nutritious diet. Some research has found that households may change their eating patterns by modifying quantity and quality of some food groups (123–125). The intake of fiber-rich vegetables, for example, have been shown to reduce calorie intake from all other sources (126, 127) and, therefore, could be a viable pathway to address the prevalence of rising chronic diseases.

While low SES is widely recognized as a driver of low vegetable consumption (12–20), the results of the stratified analysis in our study provide more granularity on behavioral patterns of low-income consumer expenditure across processing levels. The effect of family income was concentrated among the high-income group, where higher income promoted spending on fresh categories (i.e., non-processed fresh, fresh cut, fresh prepared) and impeded spending on canned vegetables. At the same time, consumers in the low-income group allocated 6.68% to non-processed fresh vegetables, which was 0.39% (*p* < 0.001) less than the high-income group. This seems to suggest that high income families are able to afford expenditure increases without sacrificing on vegetable purchases. Educational attainment, another proxy for SES, had effects similar to those of income.

In our study, educational attainment was a positive driver of expenditure share for most vegetable categories, with the exception of fresh cut that was negative. However, upon stratification of the sample into two groups as a function of SES, educational attainment had opposing effects on canned vegetable expenditure. Consumers with higher educational attainment in the low-income group allocated greater share to canned vegetables, whereas educated consumers in the high-income group devoted less share. This evidence suggests that even for low-income consumers, general education can make a positive impact on their overall vegetable intake. While general and nutrition education typically acts on the entry point of consideration to improve mental accessibility via knowledge provision or awareness-building, other dimensions of marketing can significantly impact consumer behaviors (99).

In-store variety of fruit and vegetable UPCs had heterogeneous effects across vegetable categories. Variety promoted expenditure on non-processed fresh and fresh prepared but was negatively associated with canned vegetables. In the stratified analysis, opposing effects were observed for the frozen category where variety demoted frozen expenditure share for low-income consumers, but promoted expenditure share for high-income. This provides evidence of the effect that the marketing practice of in-store variety can have on vegetable expenditure. In one of our previous studies, we also found that in-store variety, as well as in-store promotion, had strong influences on non-processed fresh vegetable expenditure (99). Commercial marketing data, typically used for guiding business practices under the “4 Ps” of marketing (product, price, promotion, and placement) (76, 77), can also be used to guide a nutrition/health mindset among management leaders in the grocery industry (99). Our present study used commercial loyalty program data, typically used for internal business intelligence and to administer consumer rewards programs, to evaluate drivers of food demand in a modern food retail system. This provides further evidence for the reciprocal linkages that can exist in industry and practice, that bridge the siloes typically persistent between marketing/business practice and nutrition/health. Again, these studies contrast with current nutrition intervention standards in which efforts remain targeted on nutrition education and lower prices (128, 129), while missing other opportunities for bolstering consumption via marketing practices like in-store promotion and diversification of fresh vegetable product UPCs. However, building a tighter link between business processes and nutrition/health is only one segment of the transdisciplinary and multisectoral convergence required to achieve significant and lasting impact on health and economic outcomes.

Convergence across the private sector, civil society, and government, as well as health and healthcare, are required to better balance supply and demand across markets, and place nutrition/health as an upfront driver of innovation and health/economic prosperity. For example, primary care clinicians (e.g., physicians, nurses, or dietitians) in healthcare settings are a relatively untapped entry-points that can influence patient/consumer behaviors (130–133). In this study for example, we see a moderate level of consumer demand for convenience, where 1.47% of expenditure share was spent on vegetable products with some degree of processing (i.e., fresh cut, fresh prepared, canned, and frozen) compared to 6.88% for non-processed fresh. This insight points to a need for innovation across varying levels of processing to meet consumer demands. However, we also saw that low-income consumers did not have as dramatic seasonal spending variation and may not be able to afford more expensive non-processed fresh foods in winter, and therefore opt for canned. This also points to a need for innovation to deliver not only convenient products, but also affordable, nutritious, and other value-add products for consumers distributed across the full SES spectrum. Convergence further extends to the health and healthcare sectors, where clinicians often act as demand generators for healthy food products. For example, community nurses were a key driver in fostering nutrition transition for mothers in Ghana (134). Convergence, however, requires an enabling policy environment and catalysis by government.

### Limitations

Beyond its scientific contribution to health-promoting nutrition, the approach used in this study has limitations to be addressed in future research. The loyalty program data only came from one retailer, although consumers may purchase food from multiple stores, retail chains, farmers' markets, or even grow their own. Although, it is notable that the retail partner for this study is one of the largest retailers in the area and our sample size was just under 300,000. Future studies should also aim to enrich consumer-level data, rather than link to broader Census demographic information, to investigate more granular and individual-level differences. In addition, retail food purchases do not necessarily reflect consumption due to food waste and, therefore, future research may consider a discovery cohort in order to trace the full cycle of purchased/homegrown vegetables and their disposal. Research in the future may also investigate alternative classifications of vegetables by their processing level, such as the NOVA food classification system (135), as well as by more granular classifications for vegetables (e.g., leafy greens, red/orange, legumes, etc.). In spite of these limitations, we believe our methodological approach is a complement to diet questionnaires and brings additional insights on vegetable expenditure patterns.

### Policy Implications

Today, most dietary guidelines tie fruits and vegetables together into one category—with the target of 400 grams/day—and do not consider differences among fruits or vegetables in terms of price, nutrient content, quality, flavor, storage, or level of processing. The outcomes of this research point to an evident need for more specific dietary guidelines that consider processing level and nutrient content for specific fruit and vegetable varieties, while also considering alternative substitutions dependent upon the socioeconomic circumstances of individuals or communities over time.

One potential solution to the budget challenge is to consider alternative types of vegetables, especially given that vegetable consumption has been significantly associated with reduced mortality from chronic diseases like cancers and cardiovascular disease (54). Miller and Knudson (136) compared the costs of eight vegetables in the form of canned, frozen, and fresh. They found that canned vegetables had comparable nutritional content when compared to fresh vegetables, and are cheaper to buy, have a longer shelf life, and use less energy for consumer storage (136). In the extreme example, household food budgets can be stretched by nearly 500% with canned green beans over fresh ones. Some frozen vegetables also provide significant savings compared to fresh vegetables. However, this message is often omitted or lost when communicating to consumers regarding healthy diet.

The results of this study highlight the positive impact that post-secondary education can have on vegetable expenditure, particularly for low-income consumers. This insight also supports much of the global development work that has focused on delivery of education as a catalyst for improving food security, community nutrition, and health interventions in both developing and developed contexts (137). In addition to general and post-secondary education, targeted nutrition education programs (e.g., skills training) have found prior success in boosting vegetable consumption (137, 138). Health literacy programs have also demonstrated positive impact on fruit and vegetable (139) and, when paired with agricultural intervention programs (e.g., home gardening education), greater synergies can be achieved (67, 69). However, the insights from this study also point to a need for curriculum expansion, with more precise messaging and content on processed vegetables, to better equip consumers with the information needed to make trade-offs and decisions regarding substitutions given their time and monetary limitations (140).

Education in its many forms, however, is only one component of potential health-promoting strategies typically used in food marketing (75, 76). Under the guide of one of the other 4 Ps of marketing, the heterogenous effects of in-store variety on expenditure across vegetable product categories demonstrate the impact that placement has on vegetable expenditure. This contributes evidence to previous research on placement that found that consumers who shop at supermarkets are more likely to consume processed foods at the expense of unprocessed foods, which is linked to lower prices paid per calorie (141). While the majority of Canadians don't consume enough fruits and vegetables (142), even high-income families could benefit by eating more frozen or canned vegetables without paying more. On the other hand, low-income families systemically spent less of their food budgets on vegetables; therefore, they face a tighter budget constraint on vegetables and are more sensitive to price changes (143). Substituting a high portion of fresh vegetable intakes with frozen or canned vegetables may be more effective for low-income families. The analytical results provided evidence of seasonal variation and that some consumers are indeed eating more non-perishable vegetables in winter to compensate for increases in their costs. These findings are in alignment with a growing body of evidence demonstrating that lowering prices for vegetables can increase their purchase and consumption (144–147). For example, price discounts on fresh vegetables during late summer, when expenditure is lowest, could incentivize their consumption and contribute to greater expenditure and intake. Future food policies may consider drawing from other marketing practices, such as advertising (99, 148), in-store price promotions (149), or healthy displays of food at the end of aisle or checkout (i.e., endcaps) (150) to incentivize vegetable expenditure over time.

Beyond the implementation of marketing strategies within any one given organization, governments can leverage their regulatory role to boost vegetable consumption (151). National retail-based subsidy programs have been proposed as a viable mechanism to boost vegetable consumption (152). However, national subsidy programs are not well-established as of yet, and the administrative mechanisms by which federal governments can implement such programs are dependent on local contexts. Effective federal- or state- level vegetable subsidy programs can leverage governmental spending power to directly influence prices, for example, with the provision of free vegetables or rebates/coupons to certain groups of the population (144–147, 152). The success of such programs rely on the voluntary participation of food retailers who also need to be willing and incentivized to participate by a sufficient exchange in value (152). Governments may also leverage taxation mechanisms to grant incentives (i.e., exemptions) for healthy foods like vegetables, or penalize unhealthy foods like sugar-sweetened beverages to generate revenue for spending on direct programs (152, 153).

Governments also play a catalytic role in the social and commercial food sector where they can support healthy food environments to curb the onset of chronic diseases tied to under- and over-nutrition (151). In their catalyst role, governments are key to gathering and sharing information, facilitating collaboration across the value chain, and the mobilizing financial resources necessary to effect change (151). For the government to fulfill each of these roles and activities, platforms and data are required to drive upstream food innovation and support decisions along the full spectrum of the value chain, from decision supports for consumers through to population-level insights for policymakers. In the geography where our study was completed (Quebec), work has already started to create a population health record for the city of Montreal where health, administrative, and retail data are integrated to yield new population health insights (154, 155). Through both their regulatory and catalytic roles, governments can accelerate convergence across the whole-of-society to create a healthier food system (156).

## Conclusion

This study provides insights on how a large sample of consumers purchase vegetables over time, and how SES and other demographic variables influence their decisions as a function of processing level. The significant seasonal variation, together with the differences in expenditure observed for low- and high-income groups, highlight the need for more targeted education on vegetable consumption and potential substitutions across processing levels. Considering that vegetable consumption is insufficient across the spectrum of SES, and that some nutritional differences are tied to processing level, greater specificity is required in nutrition guidelines across processing levels to better support yearlong trade-offs. Key opportunities lie in further linking marketing practices and data to broader agricultural, food, health, social, industrial and economic systems that structure our agri-food system, economy, and society. Governments may not only scale up present education and consumer subsidy efforts, but also act as catalysts for health-promoting investments by social and commercial enterprises across local, state, national, and global food systems. The results of this study provide insights on vegetable expenditure patterns to support health-promoting food policies and the creation of healthier food environments, while altogether considering the complexity of socioeconomic and demographic drivers at play.

## Data Availability Statement

The data analyzed in this study were obtained from a grocery retailer and are confidential. Requests to access metadata should be directed to Yun-Hsuan Wu (yunhsuan@mail.cmu.edu.tw).

## Ethics Statement

The studies are based on a set of secondary data provided by a grocery retailer. No human participants are involved in the studies, and no personally identifiable information is provided nor analyzed.

## Author Contributions

YM and LD contributed to the interpretation of the data, critical revisions, and overall supervision to the study. CM contributed to the data interpretation, wrote the manuscript, and led the revisions. Y-HW was responsible for the conceptualization of the project, study design, data analysis and interpretation, and manuscript edits. All authors critically reviewed and approved the final manuscript for submission.

## Conflict of Interest

The authors declare that the research was conducted in the absence of any commercial or financial relationships that could be construed as a potential conflict of interest.

## Publisher's Note

All claims expressed in this article are solely those of the authors and do not necessarily represent those of their affiliated organizations, or those of the publisher, the editors and the reviewers. Any product that may be evaluated in this article, or claim that may be made by its manufacturer, is not guaranteed or endorsed by the publisher.
