# Supplementary material for: Exploring Pathways of Socioeconomic Inequity in Vegetable Expenditure Among Consumers Participating in a Grocery Loyalty Program in Quebec, Canada, 2015–2017
Source: Front Public Health. 2021 Aug 2;9:634372. doi: 10.3389/fpubh.2021.634372 (PMC8365471; doi:10.3389/fpubh.2021.634372)
Supplement: Supplementary file 1 [file Data_Sheet_1.docx]

Supplementary Material

**Supplemental Figure 1.** Average vegetable expenditure share stratified by low- and high-income, month over month

**Supplemental Figure 2.** Average vegetable expenditure shares by food group stratified by low- and high-income, month over month

*Note: The definition for each subheading: (1) non-processed fresh: including whole, non-cut and non-processed vegetables and fresh herbs; (2) fresh cut: including cut vegetables, with or without dip; (3) fresh processed: including all types of salad (e.g. store-prepared salads or manufacturer-prepared salads), as well as appetizers or small plates prepared with vegetables; (4) canned vegetable: including all canned vegetable products; (5) frozen vegetable: including all frozen vegetable products.*

**Supplemental Table 1.** Characteristics of frequent consumers stratified by low- and high- income, and grocery retail stores in Quebec, Canada

|  | **Low** | | **High** | |  |
| --- | --- | --- | --- | --- | --- |
|  | **Mean** | **SD** | **Mean** | **SD** | **P-value** |
| **Consumer expenditure characteristics** |  |  |  |  |  |
| Monthly food expenditure ($) | 270.61 | 200.25 | 303.39 | 222.03 | <0.001 |
| Monthly fruits and vegetables expenditure ($) | 40.42 | 38.96 | 47.73 | 45.18 | <0.001 |
| Monthly vegetables expenditure ($) | 22.19 | 22.66 | 26.33 | 26.07 |  |
| Average monthly food expenditure share on fruits and vegetables (%) | 15.16 | 10.04 | 15.78 | 9.83 | <0.001 |
| Average monthly food expenditure share on vegetables (%) | 8.12 | 6.28 | 8.57 | 6.30 | <0.001 |
|  |  |  |  |  |  |
| **Food expenditure share for groups of vegetables, by processing level** |  |  |  |  |  |
| Non-processed fresh (%) | 6.68 | 5.68 | 7.07 | 5.70 | <0.001 |
| Fresh cut (%) | 0.15 | 0.72 | 0.19 | 0.81 | <0.001 |
| Fresh prepared (%) | 0.45 | 1.38 | 0.55 | 1.46 | <0.001 |
| Canned (%) | 0.58 | 1.28 | 0.54 | 1.20 | <0.001 |
| Frozen (%) | 0.26 | 1.09 | 0.23 | 0.94 | <0.001 |
|  |  |  |  |  |  |
| **Postal code-level neighborhood census characteristics** |  |  |  |  |  |
| Population density (/square meter) | 0.004 | 0.006 | 0.002 | 0.002 | <0.001 |
| Proportion of census families with at least one child | 38.76 | 11.67 | 51.16 | 11.14 | <0.001 |
| Proportion of the population aged 15 years and over were not married (including never married, separated, divorced, or widowed) | 48.41 | 10.61 | 35.78 | 6.37 | <0.001 |
| Proportion of single-parent families | 19.03 | 8.35 | 13.32 | 4.97 | <0.001 |
| Median family income (/$1000) | 48.17 | 9.25 | 86.54 | 25.44 | <0.001 |
| Proportion of the population aged 15 years and over with postsecondary education | 53.89 | 11.41 | 64.22 | 9.81 | <0.001 |
| Proportion of the population aged 15 years and over were employed | 53.05 | 10.99 | 64.42 | 9.37 | <0.001 |
|  |  |  |  |  |  |
| **Store characteristics** |  |  |  |  |  |
| Number of fruit and vegetable UPCs (/1000) | 1.84 | 0.25 | 1.94 | 0.21 | <0.001 |

*Note: SD = Standard Deviation; UPC = Universal Product Code*
